# Polycyclic Aromatic Hydrocarbons in Honey: A Systematic Review of Occurrence, Concentrations, and Health Risk Assessment

**DOI:** 10.3390/jox15060179

**Published:** 2025-11-01

**Authors:** Wenting Li, Surat Hongsibsong

**Affiliations:** School of Health Sciences Research, Research Institute for Health Sciences, Chiang Mai University, Chiang Mai 50200, Thailand; wenting_l@cmu.ac.th

**Keywords:** polycyclic aromatic hydrocarbons (PAHs), honey, contamination, health risk assessment, benzo[a]pyrene (BaP)

## Abstract

Polycyclic aromatic hydrocarbons (PAHs) are toxic pollutants produced by the incomplete combustion of fuels and biomass. They are highly persistent and can accumulate in the food chain. Honey, a natural product susceptible to atmospheric deposition, has recently been recognized as an important bioindicator for monitoring environmental pollution. This systematic review examined 29 articles published from 2000 to 2025 analyzing the global presence, concentrations, and potential health risks of PAHs in honey. Results showed that the sum of polycyclic aromatic hydrocarbons (ΣPAHs) concentrations in honey ranged from below the detection limit to 166.83 µg/kg. Higher levels were observed in urban and industrial areas. Seventeen studies analyzed 16 PAHs prioritized by the US Environmental Protection Agency (EPA), with benzo[a]pyrene (BaP) being the most frequently detected, a highly toxic compound. Although most samples met international food safety standards, levels exceeding European regulatory limits were detected in some areas, raising concerns about local health risks. The results of this study emphasize the need for standardized analytical methods and routine monitoring to more accurately assess the exposure risk of PAHs in honey.

## 1. Introduction

**Figure 1:**
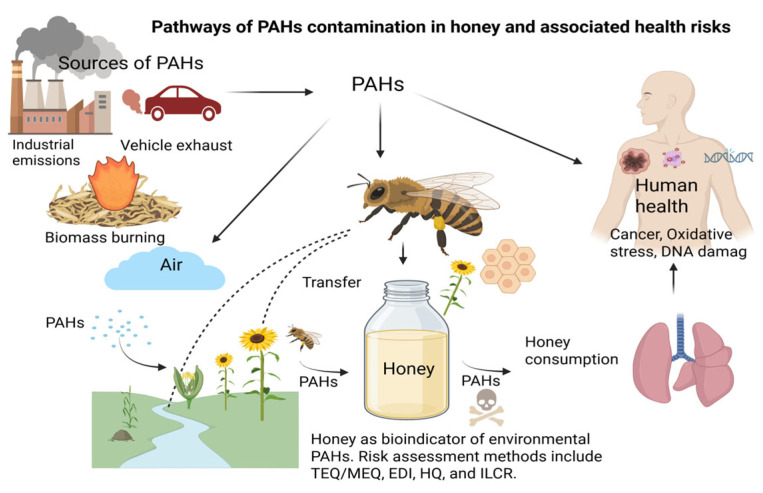
Pathways of polycyclic aromatic hydrocarbons (PAHs) contamination in honey and associated health risks.

Honey is a widely consumed natural product with recognized nutritional and therapeutic properties. It has recently gained attention as a potential bioindicator of environmental contamination due to its ability to accumulate pollutants such as heavy metals, pesticides, and polycyclic aromatic hydrocarbons (PAHs) [1,2]. Among these, PAHs have attracted special attention due to their toxicity, persistence and carcinogenicity [3]. PAHs are a class of toxic, oil-soluble organic pollutants, primarily produced by incomplete combustion of fossil fuels, biomass, and waste. These pollutants are ubiquitous in the air and can enter honey through bees collecting contaminated pollen and nectar or through the deposition of airborne particles [4]. Several PAHs—such as benzo[a]pyrene (BaP), fluoranthene (FL), and pyrene (Pyr)—have been classified by the USEPA as priority pollutants due to their toxicity and mutagenic effects [5]. Long-term exposure to PAHs may cause adverse health effects such as endocrine disruption and increased cancer risk [6]. Furthermore, emerging evidence indicates that not only parent PAHs but also their transformation products—such as oxygenated (OPAHs) and nitrated PAHs (NPAHs)—can be detected in honey. These derivatives are generated by atmospheric photochemical reactions or by microbial transformation of parent PAHs. Some may possess mutagenic and carcinogenic potency equal to or even higher than the original compounds [7,8]. Recognizing the presence of parent PAHs and modified PAHs emphasizes the need for comprehensive monitoring of honey as a food and environmental monitoring indicator. Consequently, monitoring PAH contamination in honey not only serves as an environmental assessment tool but also addresses growing concerns about food safety and public health. Figure 1 provides an overview of the environmental pathways leading to PAH contamination in honey and their associated health risks.

Despite a growing body of literature reporting PAH concentrations in honey from different regions, substantial heterogeneity remains. This heterogeneity arises from differences in analytical methods (e.g., extraction/cleanup, detection techniques), target panels (whether full EPA-16 or a subset or extended panel including OPAHs/NPAHs), and reporting formats. These differences complicate cross-study comparisons and pooled interpretations. Meanwhile, some studies have only measured the content of PAHs in honey [9,10]. Others have further used indicators such as estimated daily intake (EDI), hazard quotient (HQ), and lifetime carcinogenic risk (ILCR) to assess potential health risks [4,9]. Overall, there are significant differences in risk assessment methods in the existing literature. In addition, there is a lack of consistent standards for key assumptions such as intake rate and toxicity equivalent factors (TEFs/MEFs), which further limits the comparability of results and the reliability of meta-analysis [5,6,10,11]. Moreover, the European Union and other agencies have established maximum levels for BaP and for the sum of BaP, BbF, BaA, and Chr (PAH4) in foods. However, the application of these indicators to honey is not consistent. In many cases, the range and variability of the relevant data are also not presented in a unified way [4,12,13].

This systematic review aims to address these gaps by collating global data on the occurrence, types, and concentrations of PAHs in honey. It also evaluates methodological consistency across studies (extraction, instrumentation, target lists, and reporting) and implements a harmonized toxicity equivalency and mutagenicity equivalency (TEQ/MEQ) framework to facilitate comparable risk characterization across datasets. By integrating patterns of concentration with standardized risk assessments and discussing sources of heterogeneity and uncertainty, this review aims to inform monitoring priorities, support the development of harmonized monitoring and risk assessment guidelines, and clarify the practical relevance of honey as a sentinel matrix at the interface of food safety and environmental health. To our knowledge, this is the first systematic review that synthesizes global data on PAH occurrence and concentrations in honey. In addition, it integrates a harmonized TEQ/MEQ-based framework for risk assessment, providing new insights into honey’s role as a sentinel bioindicator of environmental pollution at the interface of food safety and environmental health.

## 2. Materials and Methods

### 2.1. Literature Search

This systematic review was conducted according to the PRISMA 2020 version of the flow chart to conduct a literature review of peer-reviewed articles on the content, concentration, and risk assessment of PAHs in honey.

This review has been registered in the PROSPERO International Prospective Register of Systematic Reviews (Registration ID: CRD420251063848). No amendments were made after initial registration.

The literature search was performed in PubMed, ScienceDirect, and Scopus. The search was conducted independently in each database, and the retrieved articles were pooled. Additional sources were searched in Embase and Google Scholar, and gray literature (e.g., conference abstracts and proceedings) was screened to minimize publication bias. The search was conducted using the following keywords and Boolean operators to find indexed articles: “polycyclic aromatic hydrocarbons” OR “PAHs” AND “honey” OR “bee product” AND “health effects” OR “health risk assessment” OR “environmental effects”.

### 2.2. Inclusion and Exclusion Criteria

Studies were considered eligible if they met the following criteria: (i) original research articles published in peer-reviewed journals; (ii) investigation of PAHs in honey or honey-related products; (iii) application of validated analytical methods, such as gas chromatography–mass spectrometry (GC–MS) or high-performance liquid chromatography (HPLC), to quantify PAH concentrations; (iv) publication in English from January 2000 to May 2025. This timeframe was selected to capture the period when standardized analytical protocols became more widely applied and regulatory awareness of PAHs increased globally.

Exclusion criteria were applied to studies that (i) focused solely on bee-related products other than honey (e.g., pollen, wax), (ii) were review articles, comments, editorials, or conference abstracts, or (iii) lacked accessible full text.

All retrieved records were imported into the reference manager EndNote to remove duplicates. Title and abstract screening were performed independently by two reviewers. Full texts of potentially eligible studies were then assessed against the inclusion and exclusion criteria. Any disagreements were resolved through discussion or consultation with a third reviewer.

### 2.3. Data Extraction

**Figure 2:**
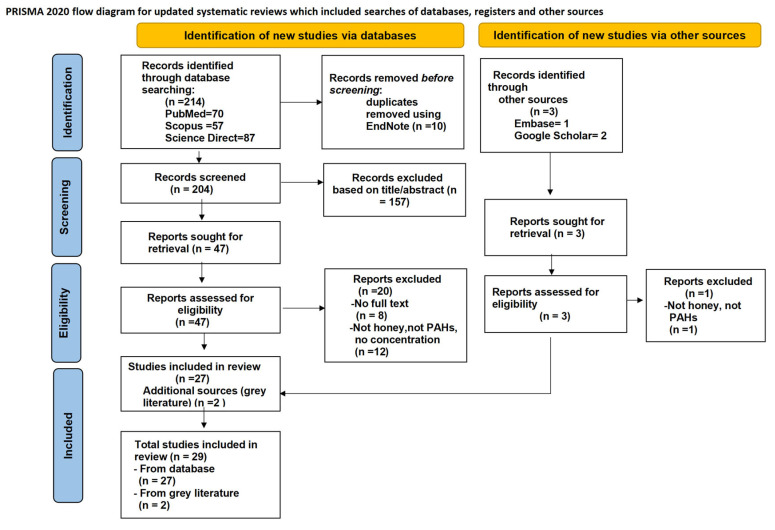
PRISMA 2020 flow diagram of study selection and inclusion process.

In this systematic review, all articles were independently assessed according to the inclusion and exclusion criteria by two researchers. They screened parameters such as title, abstract, and full text, and extracted data into a prepared format. The extracted variables included: author, year of publication, country or region, honey type (e.g., floral source, stingless bee, or western honey bee), sample size, sampling location, PAH species analyzed and their concentrations, analytical methods used, application of health risk assessment tools (e.g., estimated daily intake (EDI), hazard quotient (HQ), incremental lifetime cancer risk (ILCR)), and suspected pollution sources (e.g., urban, rural, industrial). These researchers assessed the quality of the articles according to the PRISMA 2020 guidelines [14], as shown in Figure 2.

Because the studies varied in terms of the PAH species tested, honey sources, and analytical methods, leading to significant differences in findings, this review did not include a meta-analysis. We used descriptive statistics such as mean, median, and range to summarize PAH levels and grouped and compared them according to PAH concentration level, analytical method, and risk assessment results.

### 2.4. Risk Assessment

In this review, risk assessment was performed using the benzo[a]pyrene equivalent (BaP_EQ_) approach, as outlined by Asamoah [9]. This method provides a standardized means of evaluating the carcinogenic potential of PAH mixtures. BaP_EQ_ will be estimated through two indices: the Toxicity Equivalent Quotient of BaP (TEQ_BaP_) and the Mutagenic Equivalent Quotient of BaP (MEQ_BaP_). These indices are calculated by multiplying the mutagenicity equivalence factor (MEF_i_) or toxicity equivalence factor (TEF_i_) by the sum of the concentrations of the individual PAHs, as shown in Equations (1) and (2) [15].

(1) $$TEQ_{BaP}=\Sigma \left(TEF_{i}\times C_{i}\right)$$

(2) $$MEQ_{BaP}=\Sigma \left(MEF_{i}\times C_{i}\right)$$

where C_i_ is the concentration of each PAH, and TEF_i_ or MEF_i_ represents its respective toxicity or mutagenic equivalent factor [10]. The TEF_i_ values for BaA, BaP, BbFlu, BkFlu, Chr, DahA, and IndP were 0.1, 1, 0.1, 0.01, 0.001, 1, and 0.1, respectively [11]. The MEF_i_ values for BaA, BaP, BbFlu, BkFlu, Chr, DahA, and IndP were 0.082, 1, 0.25, 0.11, 0.017, 0.290, and 0.310, respectively [11]. The BaP equivalent dose was calculated based on the following Equation (3) [9].

(3) $$BaP_{EQ}=\frac{\left(TEQ\;\mathrm{or}\;MEQ\right)\times IR\times EF\times ED}{BW\times AT}$$

Based on Equation (3), IR represents the daily ingestion rate of honey, set at 10 g/day for adults according to the U.S. EPA/WHO default value [11]. EF is the exposure frequency (350 days/year), and ED is the exposure duration (30 years for adults). According to the USEPA Risk Assessment Guidance for Superfund (1989) [16] and the Exposure Factors Handbook (2011) [11], a 30-year exposure duration is a standard default assumption for chronic adult dietary or residential exposure, representing a screening-level estimate. However, we acknowledge that the U.S. EPA also recommends applying a 70-year lifetime as the averaging time (AT) for carcinogenic risk assessment, which would yield more conservative risk estimates. BW represents body weight (70 kg for adults) [16]. For non-carcinogenic risk, AT is calculated as EF × ED = 10,950 days, whereas for carcinogenic risk, AT corresponds to a lifetime of 70 years (25,550 days) [11]. The definitions and assumptions for all variables are summarized in Table 1. The final cancer or mutagenic risk was estimated using Equation (4) [10].

(4) $$\mathrm{Cancer}\;\mathrm{or}\;\mathrm{mutagenic}\;\mathrm{risk}=SF_{BaP}\cdot BaP_{EQ}$$

where $SF_{BaP}$ is the oral carcinogenic slope factor for BaP (7.30 mg/kg per day) [10]. According to the U.S. Environmental Protection Agency’s guidelines, cancer risk (CR) values greater than 1 × 10^−4^ are considered high risk. Values between 1 × 10^−6^ and 1 × 10^−4^ are considered acceptable, while values less than 1 × 10^−6^ are considered negligible [16].

### 2.5. Limitations of the Risk Assessment

This risk assessment provides screening-level estimates and should be interpreted with caution. Several uncertainties may affect the calculated TEQ/MEQ and BaP-equivalent carcinogenic risk values:

Analytical measurement error: Differences in extraction recoveries, detection limits, and instrument precision across studies may introduce potential quantitative bias.

Exposure assumptions and duration: Standard default values from the United States Environmental Protection Agency (USEPA) were used for intake rate (IR), exposure frequency and duration (EF/ED), and body weight (BW), which may not be representative of all populations. In particular, the use of a 30-year exposure duration (ED) as a screening-level default may underestimate long-term cancer risks compared with a full 70-year lifetime scenario.

Toxic equivalency factors (TEF/MEF): Slightly different TEF_i_ and MEF_i_ values were reported across studies, which may increase the uncertainty of the final TEQ/MEQ.

Inter-study heterogeneity: Differences in polycyclic aromatic hydrocarbon (PAH) target lists, sampling seasons, and geographic origins may affect the representativeness of the pooled estimates.

Because most included studies reported only point estimates or concentration ranges, rather than the full sample-level variance, formal derivation of analytical variances was not possible. As a result, statistical confidence intervals could not be generated. Therefore, we qualitatively discuss potential sources of bias and key uncertainties. The calculated cancer risk values should be interpreted as approximate risk levels rather than precise population-wide values. To avoid overinterpretation, we focus on relative comparisons (across regions and study designs) rather than exact absolute risks, and we highlight which assumptions have the greatest impact on risk rankings. These limitations should be considered when interpreting the findings of this review.

## 3. Results

### 3.1. Processing the Systematic Review

The search strategy applied in the current study is shown in Figure 2, according to the PRISMA flow chart. A total of 214 articles were retrieved from international databases, including Scopus (*n* = 57), PubMed (*n* = 70), and ScienceDirect (*n* = 87), covering the period from 1 January 2000 to 31 May 2025. At the start of the study, 10 duplicate articles were excluded. Only 204 articles were considered relevant based on their titles, and 157 articles were excluded because their abstracts were irrelevant. The remaining 47 articles were evaluated based on their full-text content; 12 articles were excluded because they did not contain honey, PAHs, or specific concentrations; and 8 articles were excluded because the full text was not available. In addition, 2 articles were retrieved from the gray literature search, resulting in a total of 29 full-text articles included in the review.

### 3.2. Characteristics of Reviewed Studies

Most of the 29 included studies were published in recent years, with the highest number in 2023 (*n* = 8), followed by 2022 (*n* = 4) and 2017 (*n* = 4), indicating growing research interest in PAH contamination in honey.

**Figure 3:**
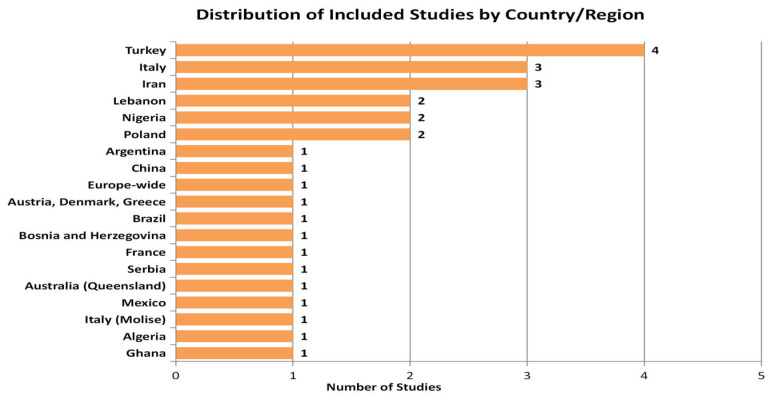
Distribution of included studies by country or region.

The reviewed literature demonstrates broad geographical coverage. As indicated in Figure 3, Turkey was the most frequently studied country (*n* = 4), followed by Italy and Iran (*n* = 3 each), with additional contributions from Lebanon, Poland, Nigeria, and other regions.

Regarding sample types, most studies analyzed commercial honey [8,17], while others included blossom honey [1,3], or honey from specific floral origins (e.g., Codonopsis) [18], or blank matrix honey used for laboratory validation [19]. Some studies also included bee samples or honey from managed colonies [20]. However, only honey-specific results were extracted for this review.

Analytical methods differed among the included studies. GC–MS and GC–MS/MS are the most commonly used techniques for detecting PAHs [21,22,23]. QuEChERS extraction methods [24,25], often combined with SPME or GC–FID, were reported in several studies [2,26]. A few studies employed advanced detection approaches such as gas chromatography-selected ion monitoring–mass spectrometry (GC–SIM–MS) or used covalent triazine frameworks to improve extraction efficiency [2]. Key methodological parameters of the included studies, including extraction protocols, detection techniques, and LOD/LOQ values, are summarized in Table 2 to illustrate sources of heterogeneity.

Based on the above summary of analytical techniques, this subsection further evaluates the floral origin of honey to examine its potential association with PAH concentrations. Among the 29 included studies,1 explicitly analyzed unifloral honeys (honeys (e.g., Wang, W. et al. [36]), 17 focused on multifloral honeys, 5 studied mixed types (both unifloral and multifloral), and 6 did not report floral origin (NR) [1,7,23]. Based on the available data, no consistent association was observed between ΣPAH content and honey floral type. For example, some multifloral honeys had the highest ΣPAH concentrations (Lebanon, 166.83 µg/kg [2]), while others reported the lowest ΣPAH concentrations (Italy, 0.16 µg/kg [31]). This suggests that local environmental pollution sources, such as traffic, biomass burning, and industrial activities, are the main drivers of PAH contamination in honey, rather than floral species [28,30]. Previous studies on plant-specific nectar chemical properties have also shown that the floral species itself is unlikely to directly affect PAH accumulation [28].

### 3.3. PAH Species Detected

The 29 studies included in this review analyzed a diverse range of PAHs, with notable differences in both the number and specific compounds assessed across studies. A total of 17 studies (58.6%) specifically targeted all 16 priority PAHs designated by the USEPA, which are widely regarded as the standard reference for evaluating environmental pollutants and food contaminants [26,29]. These studies typically employed standardized analytical techniques such as GC–MS or GC–MS/MS, HPLC, and QuEChERS based extraction protocols to ensure accurate detection and quantification [2,18].

The remaining 12 studies either focused on partial subsets of the EPA 16 PAHs or extended their analytical scope to include oxygenated PAHs (OPAHs), nitrated PAHs (NPAHs), or alkylated derivatives [20,28,29]. These studies were excluded from the EPA 16-complete count because they did not report all 16 compounds individually, omitted specific PAHs (e.g., acenaphthylene (Ace) or indeno[1,2,3–cd]pyrene), or did not explicitly confirm that the full EPA panel was assessed [18,25].

Among individual PAHs, BaP was the most frequently detected compound, identified in 26 out of 29 studies. This highlights its pivotal role in health risk assessment and regulatory oversight [2,19,34]. Pyr and Chr were each detected in 24 studies [18,20,29]. Phenanthrene (Phe) and fluorene (FLu) were reported in 22 studies [7,23,25], while naphthalene (Nap) and benzo[a]anthracene (BaA) appeared in 21 studies [2,4,29]. Fluoranthene (FL) was found in 19 studies [22,23,27], and acenaphthene (Acy), benzo[b]fluoranthene (BbF), and indeno[1,2,3-cd]pyrene (IcdP) were identified in 18, 18, and 17 studies, respectively [2,27,29]. These compounds, mainly composed of 3 to 5 ring structures, are usually related to incomplete combustion of organic matter, exhaust emissions and industry. The combination of environmental stability and measurable presence in foods like honey is largely attributed to their mid-range molecular weights. Consequently, they are frequently included in targeted residue analyses for risk characterization purposes [2,26].

While BaP was the most frequently detected high-toxicity PAH, the highest reported concentrations across studies often corresponded to mid-weight PAHs such as Naph, up to 33.3 µg/kg in Lebanon [2], FLu, up to 33.03 µg/kg in Lebanon [2], FL, up to 5.91 µg/kg in Italy [30], and Phe, up to 10.33 µg/kg in Lebanon [2]. In addition, more toxic but less commonly reported compounds were also identified at elevated levels, including DahA, up to 11.4 µg/kg in Nigeria [9] and IcdP, up to 118.25 µg/kg in Iran [29].

These findings highlight an important distinction. While BaP dominates in terms of detection frequency and toxicological importance, the maximum concentrations in honey samples were more often driven by Naph, FLu, FL, Phe, and certain high-molecular-weight PAHs. This dual pattern—frequent detection of BaP but peak concentrations of other PAHs—underscores the need to consider both occurrence frequency and concentration magnitude when characterizing risk.

Furthermore, some studies expanded their analytical scope beyond the EPA’s 16 priority PAHs, investigating up to 40 individual compounds. These included OPAHs, such as 9,10-anthraquinone reported up to 103 µg/kg in Argentina [8], and NPAHs, such as 1-nitronaphthalene [20,29], which are increasingly recognized for their toxicological relevance. Although researchers do not usually test these PAH derivatives, actual studies have shown that they contribute significantly to honey contamination in industrial and heavily polluted areas [20,28].

**Figure 4:**
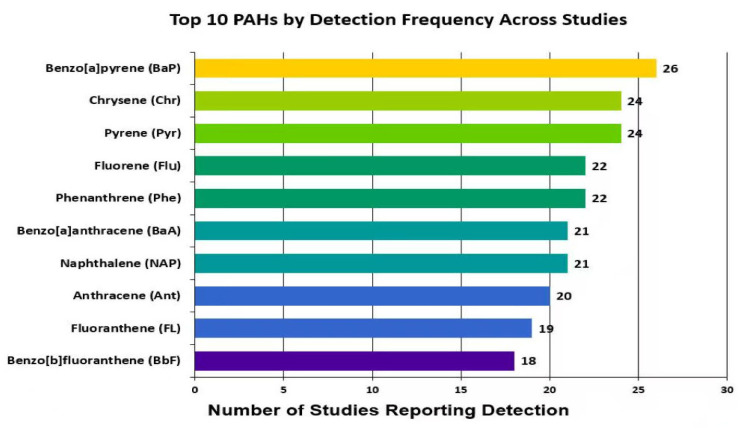
Top 10 PAHs by Detection Frequency Across Studies.

Overall, the detection trends underscore the continued relevance of the EPA’s 16 priority PAHs as a globally recognized benchmark for environmental monitoring and regulatory comparison. These compounds are among the most toxic and environmentally persistent PAHs, which is why they serve as the foundation for risk assessment. At the same time, recent studies have expanded their scope to include additional PAH compounds—such as OPAHs, NPAHs, and alkylated derivatives. These groups are increasingly recognized for their toxicological potency and environmental relevance. By considering both the EPA 16 priority PAHs and the broader PAH spectrum, this review provides a more comprehensive picture of honey contamination and its potential health implications. Figure 4 further illustrates the detection frequencies of the ten most commonly reported EPA priority PAHs, providing a valuable reference for prioritizing target compounds in future monitoring.

### 3.4. PAH Residues in Honey

The concentration of total polycyclic aromatic hydrocarbons (ΣPAHs) in honey varied substantially across studies and countries, with reported mean values ranging from as low as 0.01 µg/kg [29] to as high as 166.83 µg/kg [2]. Most studies focused on the 16 priority PAHs defined by the USEPA, which are widely used as standard indicators for environmental pollution and health risk assessment [10]. However, several investigations extended their analyte scope to include additional PAHs, such as nitrated (NPAHs) and oxygenated PAHs (OPAHs), to better capture the full spectrum of contamination and toxicological relevance [20,28,29].

The highest reported mean concentration of total PAHs (ΣPAHs) was observed in a study from Lebanon (mean = 166.83 µg/kg), based on QuEChERS–SPME extraction combined with GC–MS/MS detection [2]. This exceptionally high level may reflect either intense local contamination or the use of highly sensitive analytical methods. In contrast, European samples generally exhibited lower ΣPAH levels. For example, mean concentrations in Poland and Italy were reported at 1.03 µg/kg [34]and 0.16 µg/kg [31], respectively. Turkish honey showed moderate contamination, with reported means ranging from 1.71 µg/kg to 10.12 µg/kg, depending on regional origin and sampling location [32].

In several studies, individual honey samples exceeded 10 µg/kg of ΣPAHs, surpassing typical background levels. Notably, extremely high concentrations were reported in Argentina (up to 103 µg/kg, including oxygenated PAHs) [8], Brazil (up to 23.3 µg/kg in stingless bee honey) [24], and Iran (up to 118.25 µg/kg of indeno[1,2,3–cd]pyrene) [29]. These extreme values likely reflect point-source pollution or site-specific anthropogenic activities, such as open biomass burning, vehicular emissions, or industrial discharge.

According to Commission Regulation (EU) No 835/2011, the maximum permissible level of BaP in foodstuffs is 1.0 µg/kg [12]. However, a study in Bosnia and Herzegovina found that the concentration of benzo[a]pyrene in honey exceeded the EU limit, reaching 6.12 μg/kg [12,26]. While most studies did not explicitly evaluate risk based on WHO or EU benchmarks, several conducted estimated daily intake (EDI) and margin of exposure (MOE) assessments. These assessments generally concluded low health risk when MOE > 10,000 and HI < 1 [13]. Likewise, evaluations based on the hazard index (HI) followed World Health Organization (WHO) principles, with HI values < 1 suggesting an acceptable level of risk [6].

The differences in PAH levels in honey between countries are influenced by multiple factors. These include regulatory laws, actual environmental pollution conditions, as well as the sample preparation protocols and analytical methods adopted in each study. Variations in extraction procedures (e.g., QuEChERS, solid-phase extraction) and instrumental techniques (e.g., GC–MS, GC–MS/MS, HPLC) may lead to differences in sensitivity, recovery, and detection limits, thereby affecting the comparability of reported concentrations. For example, the European Union has set an annual average limit of BaP in air of 1.0 ng/m^3^ under Directive 2004/107/EC to avoid long-term exposure to this carcinogenic compound [39]. Although the detection methods used by different research teams are different, the academic community has increasingly recognized the role of honey in environmental pollution detection due to its wide availability and ability to absorb oily pollutants.

As summarized in Table 3, the highest mean ΣPAH concentration was observed in Lebanon (166.83 µg/kg; calculated as the sum of the nine PAHs quantified by Al–Alam et al. [2]). f High levels were also found in urban samples from Turkey (10.12 µg/kg) [35] and in honey from Argentina, where total PAHs including derivatives reached up to 103 µg/kg [8]. In contrast, markedly lower concentrations were reported in Italy (0.16 µg/kg) [31], Austria and Iran, where values as low as 0.01 µg/kg were recorded [20,29]. These findings reflect that PAH pollution levels may be affected by local environmental conditions, pollutant emission sources, and policy enforcement.

### 3.5. Health Risk Assessment

Among the 29 included studies, 10 reported their own health risk assessments based on PAHs in honey or bee-related matrices. These assessments utilized methodologies such as BaP-based individual risk thresholds, hazard quotients (HQ), TEQ_BaP_, and incremental lifetime cancer risk (ILCR) models, primarily derived from USEPA and the EU guidance [10,13].

As summarized in Table 4, studies conducted in Serbia [4], Iran [19], and Nigeria [9] reported moderate health risks, primarily attributed to BaP concentrations exceeding 1 µg/kg or incremental lifetime cancer risk (ILCR) values approaching or surpassing the benchmark of 1 × 10^−4^ [4]. In contrast, studies from Algeria [7], Poland [34] and Ghana [38], indicated generally acceptable risk levels, with hazard quotient (HQ) or BaP concentrations remaining below regulatory thresholds. Additionally, Murcia–Morales et al. assessed TEQ_BaP_ in bee tissue, providing valuable but indirect insights into potential health risks associated with honey consumption [20].

In parallel, as presented in Table 4, standardized health risk estimations were performed across all 29 studies based on TEQ_BaP_ and MEQ_BaP_, using the USEPA oral slope factor for BaP (7.3 mg/kg/day) [10]. Based on these estimates, 19 of the 29 studies (65.5%) had a cancer risk (CR) greater than 1 × 10^−4^ and were therefore classified as “risk”. In contrast, only a few studies—specifically those conducted in Iran [32], France [28]and Argentina [8]—reported CR values estimated from TEQ_BaP_ or MEQ_BaP_ below the 1 × 10^−4^ benchmark, indicating acceptable or negligible risk levels.

These findings confirm that while some studies independently identified health risks, the majority of calculated risks based on standardized TEQ_BaP_ and MEQ_BaP_ models revealed indicated elevated potential carcinogenic risk, especially in samples from urban or industrial regions in Africa, Latin America, and parts of Asia. To place these estimates into proper context, the methodological limitations and inherent uncertainties of the risk assessment are discussed below.

Current characterization of cancer risk provides only screening-level estimates. Most included studies reported point estimates or concentration ranges without full sample-level variance. This limitation makes it impossible to calculate provisional tolerable weekly intakes (PTWIs) with statistical confidence intervals.

Therefore, we focus on TEQ/MEQ-based cancer risk screening (e.g., negligible, acceptable, or high-risk categories) and provide a qualitative discussion of the main sources of uncertainty. These include analytical variability, exposure assumptions, and heterogeneity in TEF/MEF values. For example, detection limits differ among GC–MS methods, daily honey intake estimates vary across regions, and some studies used alternative TEF/MEF sets recommended by USEPA or EFSA.

At present, there are no internationally accepted reference values for the PTWI of PAHs in honey. In addition, the available data are insufficient to derive robust PTWIs with statistical confidence intervals. Future monitoring that incorporates detailed individual intake data will make it possible to establish PTWIs supported by confidence intervals.

## 4. Discussion

This study systematically reviewed 29 research papers on PAH pollution in honey from 2012 to 2024, with a focus on analyzing the pollution characteristics and health risks of PAHs in honey samples from different regions. The analysis results indicate that due to differences in detection methods (such as GC–MS and HPLC) and analysis targets, there is significant heterogeneity in the reported PAH concentrations among studies (ND–166.83 μg/kg). This difference highlights the importance of establishing a unified analysis method, while also indicating the need to develop standardized health risk assessment frameworks to accurately evaluate the potential impacts of PAH pollution on food safety and public health.

### 4.1. Comparison with Previous Findings

The reported ΣPAH concentrations varied widely—from ND to 166.83 µg/kg—reflecting heterogeneous environmental conditions, diverse honey origins, and differing analytical methodologies [2,29]. Notably, honey samples from Lebanon exhibited the highest ΣPAH levels, exceeding values reported in Turkey, Ghana, and several European countries [26,32]. Several contextual factors may help explain the unusually high ΣPAH concentrations observed in Lebanese honey (166.83 µg/kg) [2]. First, Lebanon has heavy road traffic and frequent diesel generators, which are a recognized source of atmospheric PAHs [40,41]. Second, in some rural and peri-urban areas, household heating and open burning of waste are common, leading to elevated PAH levels in the environment [42,43]. Third, topography and meteorological conditions in parts of Lebanon may also be contributing factors. Lebanon’s terrain is predominantly mountainous, with many valleys, such as those with poor ventilation, that favor the accumulation of combustion aerosols [44,45]. Finally, differences in analytical methods compared to other studies may also play a role. For example, the use of a sensitive QuEChERS–SPME/GC–MS/MS protocol [2] may have enhanced the detection of a wider range of PAHs. These factors combined may explain the unusually high levels of PAHs reported in Lebanon. These findings are consistent with prior regional studies indicating elevated airborne PAHs in areas affected by vehicular traffic, biomass burning, or industrial activities [19,20].

In contrast, countries such as Poland and Iran reported lower mean concentrations (typically < 1 µg/kg), suggesting that honey produced in cleaner or more regulated environments may contain fewer PAH contaminants [3,18]. Such differences underscore honey’s potential as a bioindicator of ambient PAH pollution, as also evidenced by studies analyzing bees, pollen, and propolis as environmental matrices [20,28]. Importantly, while most honey samples fell below the EU regulatory threshold for BaP (1 µg/kg) and PAH4 (10 µg/kg) [12], isolated cases such as those reported in Bosnia and Herzegovina [26] and Nigeria [9] exceeded these safety limits, raising potential concerns for local dietary exposure.

### 4.2. Consistency in Target Compounds

Among the 29 included studies, 17 (58.6%) explicitly analyzed the full suite of EPA 16 priority PAHs, providing the most consistent basis for inter-study comparison [18,19,26]. However, inconsistency remains a key challenge. 12 studies either adopted expanded PAH panels, including oxygenated (OPAHs) and nitrated PAHs (NPAHs), or omitted key EPA compounds such as Acy or IcdP, thereby limiting comparability across datasets [7,20]. Within the EPA 16 list itself, high-toxicity compounds also deserve special attention. BaP, for example, was reported in 26 studies and frequently used as a proxy for carcinogenicity and compliance monitoring, in line with the European Commission’s regulatory limit of 1.0 µg/kg for foodstuffs under Regulation (EU) No 835/2011 [12]. Notably, one Iranian study reported BaP concentrations up to 11.63 µg/kg, far exceeding this threshold and underscoring the need for targeted monitoring and stricter regulatory oversight in affected regions [19]. Beyond BaP, other high-toxicity PAHs such as Chr, BbF, and DahA were also reported in multiple studies [4,9,19,26], though generally at lower frequencies and concentrations. These compounds, recognized as probable human carcinogens by the International Agency for Research on Cancer (IARC) [46], merit closer attention in future biomonitoring and regulatory assessments.

Notably, OPAHs and NPAHs are increasingly recognized as emerging contaminants of growing toxicological and environmental concern. Their inclusion in recent studies reflects a broader scientific effort to capture the full spectrum of PAH-related risks, highlighting the need for harmonized monitoring frameworks that integrate both conventional and derivative PAHs.

Some studies have shown that OPAHs can arise from photooxidation of parent PAHs in the atmosphere or through microbial transformation during honey storage, and their mutagenic and carcinogenic activities may equal or exceed those of the parent compounds [47,48]. NPAHs, by contrast, are primarily formed through nitration of parent PAHs in the presence of nitrogen oxides (NOx) and oxidants. They are widely recognized as potent mutagens in urban aerosols [49]. The detection of these compounds in honey indicates contributions from both direct combustion sources (e.g., vehicle exhaust, biomass burning) and secondary atmospheric reactions.

Whether these transformation products are included in monitoring will significantly affect the TEQ/MEQ and health risk assessment results. Therefore, establishing a unified testing framework that integrates both parent and modified PAHs is essential for robust food safety evaluations and environmental exposure assessments.

### 4.3. Risk Assessment and Safety Implications

Given the well-established association between PAHs and cancer, their detection in honey has undoubtedly raised significant public health concerns. Of the 29 studies included, 10 used established methods to conduct explicit health risk assessments, such as BaP-based thresholds, EDI, HQ, TEQ, and ILCR. To assess potential health risks, many studies adopted threshold values suggested by regulatory bodies such as the USEPA and EFSA [5,10].

As shown in Table 4, studies from Algeria [7], Turkey [32], Poland [34], and Ghana [17] generally reported acceptable or low risk, with ILCR or HQ values below regulatory concern thresholds. In contrast, moderate to high risks were noted in samples from Nigeria [9], Iran [19] and Brazil [24], primarily due to elevated BaP concentrations exceeding the EU threshold of 1.0 µg/kg or elevated TEQ values.

Complementing these findings, Table 5 presents standardized TEQ- and MEQ-based cancer risk estimates for all 29 studies. Using the USEPA’s oral slope factor for BaP (7.3 mg/kg/day), the calculated cancer risk (CR) values revealed that 19 studies (65.5%) exceeded the threshold of 1 × 10^−4^, thus falling into the “risk” category. These elevated CR values were especially prevalent in studies conducted in Lebanon [2], Nigeria [9], Bosnia and Herzegovina [26], and Turkey [35]. Conversely, only a limited number of studies—particularly those from Iran [19], France [28], and Argentina [8]—demonstrated acceptable or negligible risk levels, with CR values below the 1 × 10^−4^ benchmark.

Overall, this study systematically analyzed existing literature on health risk assessment methods for PAHs in honey. The study found that, while numerous studies have attempted to assess PAH exposure risk, significant discrepancies exist in the assessment criteria used. These methodological inconsistencies complicate cross-study comparisons and highlight the urgent need for a unified approach to assessing PAH risk in the diet. Therefore, this study innovatively employed standardized TEQ and MEQ assessment systems to systematically quantify the health risks of PAH contamination in honey, providing an important basis for establishing a unified risk assessment framework. Although honey itself is not a major dietary source of PAH intake, its contamination patterns provide valuable insights into environmental exposures, reinforcing its role as a sentinel biomonitor of atmospheric pollution. These findings further emphasize the need to strengthen honey contamination monitoring and develop regionally adapted risk management measures.

To better understand these estimates, it is necessary to emphasize the limitations of this study’s risk assessment method and its inherent uncertainties. As previously discussed in Section 2.5, uncertainties arise from analytical sensitivity, intake assumptions, and differences in TEF/MEF values. This paper focuses on the impact of these uncertainties on the interpretation of the results. For example, regions with limited surveillance data (such as parts of Africa and South Asia) may lead to underestimates or overestimates of cancer risks. Seasonal heating or open burning can cause short-term PAH concentration peaks, and a single sampling is often unable to fully reflect this change. In addition, differences in TEF/MEF parameters used by different studies may also change the risk ranking of individual studies.

### 4.4. Comparison of PAHs in Honey with Other Food and Environmental Sources

PAHs have been extensively studied not only in honey but also in other foods and environmental media due to their widespread distribution and carcinogenicity, particularly in foods such as bacon, cooking oils, cereals, and tea, where PAH concentrations are consistently higher than typical values in honey.

For example, Nanaobi et al. studied the contamination levels of PAHs in different fish species, with concentrations ranging from 87.19 μg/kg to 180.19 μg/kg, with the highest concentration in mackerel, and concluded that the increased PAH concentrations were mainly due to traditional smoking and drying [50].

Similarly, Kim et al. reported that smoked and grilled fish and meat products in Korea contained PAH concentrations several times higher than those found in honey, and highlighted that the associated cancer risk was not negligible [51]. Significant differences were also observed in the PAH contamination levels of edible oils. According to Wu et al., the mean concentration of ΣPAHs in olive oil was 5.7 μg/kg, in sesame oil it was 13.2 μg/kg, and in sunflower oil it was 4.9 μg/kg, with sesame oil having the highest levels. These findings highlight the fact that PAHs are formed in edible oils through thermal processing [52].

Cereal foods also contain measurable levels of PAHs, but the concentrations are generally lower than those found in smoked or high-fat products. Einolghozati et al. reported that mean ΣPAH levels in cereals ranged from 2.8 to 13.5 μg/kg, depending on the type of cereal and processing method, with an overall weighted mean concentration of 6.27 μg/kg for the included studies, with high-temperature processing such as baking and extrusion being identified as the main contributors to PAH formation [53].

Tea is another food group for which PAH contamination has been documented. Ahmadi et al. reported that the mean ΣPAH levels in tea ranged from 2.15 to 23.1 μg/kg. Black tea and smoked tea had the highest levels, while non-smoked varieties such as green and white tea had significantly lower PAH residues, which is closely related to the burning and drying methods used during processing [54].

In addition to food, environmental components such as air, dust, and surface water are also major sources of human exposure to PAHs. Notably, in densely populated urban environments, even in countries with relatively good overall emission controls, atmospheric concentrations of BaP are generally higher than the 1 ng/m^3^ limit set by EU Directive 2004/107/EC [12]. For example, average BaP concentrations ranging from 4.2 to 15.6 ng/m^3^ have been reported in high-traffic areas of India and China, significantly exceeding those typically inferred from indirect exposure via honey or other bee-related matrices [37,40,55]. In addition, global emission inventories indicate that atmospheric PAHs remain a serious environmental burden, with Asia accounting for the largest share of global emissions [56].

In terms of health risk assessment, EDI and ILCR values for smoked meats, edible oils, and certain teas are often >1 × 10^−3^, notably higher than those reported in honey studies [11]. Although cereal products tend to have lower Mean ΣPAH value, some processed items still surpass safety thresholds for dietary exposure [53].

Overall, these comparisons suggest that honey serves primarily as a bioindicator of ambient PAH contamination, rather than as a primary source of dietary intake. Its sensitivity to atmospheric pollutants, particularly through deposition and foraging, makes it a practical and informative passive environmental monitoring medium. This is especially relevant in settings where formal air quality assessments are limited or impossible.

### 4.5. Strengths and Limitations

Strengths of this review include its systematic and transparent approach, including standardized data extraction, harmonized PAH concentration units, and systematic grouping of studies according to analytical methods. These efforts improved the consistency of the datasets and made the quantitative synthesis of the findings more robust and coherent.

Although this review provides some important insights, it also has certain limitations. Many of the included studies did not provide essential methodological details, such as limits of detection (LOD), recoveries, and quality assurance or control protocols. The absence of this information may affect the credibility and reproducibility of their reported results. In addition, the honey samples included in the reviewed studies exhibit significant heterogeneity in terms of variety and geographical origin. This diversity limits direct comparability across studies and complicates pooled analysis. Furthermore, despite South Asia and North America’s significant role in the global honey industry, data from these two regions remain insufficient, creating notable information gaps.

### 4.6. Implications and Future Directions

The findings of this review demonstrate that honey can serve as a reliable sentinel for assessing environmental PAH contamination, provided that consistent methods are applied for sampling and analysis. Evidence from the reviewed studies shows that variations in extraction protocols, detection techniques, and target compound lists directly affect the reported concentrations and the outcomes of risk assessments. These discrepancies highlight the urgent need for harmonized monitoring strategies.

Regulatory agencies and researchers should therefore prioritize several actions. First, routine monitoring of BaP and other high-priority PAHs should be promoted, especially in areas with documented environmental risk factors, such as heavy traffic, biomass burning, or industrial activity. Beyond these immediate priorities, broader methodological harmonization is also essential.

To translate these findings into practical monitoring and regulatory actions, a unified analytical and risk assessment framework is needed. Based on the observed methodological heterogeneity, we propose a unified framework for future monitoring and risk assessment of PAHs in honey. This framework comprises three key elements:

- Sampling: Adopt a standardized and transparent protocol for site selection (urban/rural/industrial), sample size, and storage, ensuring representativeness and minimizing contamination.
- Analytical Methods: Adopt a unified QuEChERS–GC–MS/MS or GC–MS protocol, adhere to agreed-upon quality assurance/quality control standards (limits of detection/quantification, recovery), and mandate reporting of ΣPAHs, BaP, and PAH4.
- Risk Assessment: Adopt internationally recognized TEQ/MEQ procedures with explicit quantification of uncertainties (e.g., confidence intervals, scenario analysis), and, where feasible, derive a PTWI.

Integrating these steps will enable consistent comparisons across regions, facilitate meta-analyses, and provide a solid foundation for global food safety standards.

At the same time, the scope of monitoring should be broadened. Several included studies have shown that oxygenated and nitrated PAHs (OPAHs and NPAHs) occur in honey, often at levels comparable to or exceeding those of parent compounds. These derivatives are known to possess strong mutagenic or carcinogenic potential and are increasingly recognized as emerging contaminants. Their inclusion in future monitoring programs is essential for a more complete risk characterization.

Finally, future work should aim to establish benchmark ranges of PAH concentrations in honey from different regions and production systems. Such reference values would provide regulators with a clearer basis for evaluating compliance and would strengthen honey’s role as a cost-effective environmental bioindicator. By integrating standardized methods, harmonized risk assessment frameworks, and an expanded analyte scope, honey monitoring can better inform public health protection and environmental policy.

## 5. Conclusions

Through a systematic analysis of existing literature, this study found that PAH pollution in honey exhibits distinct regional characteristics. Although most studies focused on the 16 priority PAHs listed by the USEPA, there were significant differences in reported contamination levels and risk assessment results due to different detection techniques and sample collection locations. These differences in analytical scope and methodology complicate cross-study comparison and limit the establishment of a unified global database. Even so, the evidence supports honey as a sensitive bioindicator of PAHs, especially for contamination arising from combustion sources.

Risk assessments in most studies suggested low concern for consumers, with HQ and ILCR values remaining below international thresholds. However, exceedances of the EU BaP limit (1 µg/kg) were recorded in some countries, including Nigeria and Bosnia and Herzegovina. These findings indicate localized contamination episodes and highlight the importance of continued surveillance in high-risk areas.

Future research should address three priorities. First, establish internationally harmonized detection and risk assessment standards to improve comparability. Second, expand the analytical scope to include oxygenated, nitrated, and alkylated PAHs, which may also contribute significantly to health risks. Third, investigate temporal and regional exposure trends to refine the role of honey as a cost-effective sentinel for food safety and environmental pollution monitoring.

## Figures and Tables

**Table 1:** (**a**). Assumptions and parameters used in the PAH health risk assessment model. (**b**). Toxicity equivalence factors (TEFs) and mutagenicity equivalence factors (MEFs) applied for selected PAHs.

**(a)**			
**Variable**	**Definition**	**Value/Unit**	**Ref.**
IR (Ingestion rate)	Daily honey intake	10 g/day (WHO default)	[11]
EF (Exposure frequency)	Number of exposure days/year	350 days/year	[16]
ED (Exposure duration)	Period of exposure for adults	30 years (screening); 70 years (lifetime, carcinogenic risk)	[16]
BW (Body weight)	Average adult weight	70 kg	[16]
AT (Averaging time)	ED × 365 (non-carcinogenic); 70 years × 365 (carcinogenic)	10,950 days/25,550 days	[16]
SF_BaP	Oral carcinogenic slope factor for BaP	7.30 (mg/kg/day)^−1^	[11]
**(b)**			
**Compound**	**TEF**	**MEF**	**Ref.**
Benzo[a]anthracene (BaA)	0.1	0.082	[11]
Benzo[a]pyrene (BaP)	1.0	1.0	[11]
Benzo[b]fluoranthene (BbF)	0.1	0.25	[11]
Benzo[k]fluoranthene (BkF)	0.01	0.11	[11]
Chrysene (Chr)	0.001	0.017	[11]
Dibenzo[a,h]anthracene (DahA)	1.0	0.290	[11]
Indeno[1,2,3–cd]pyrene (IcdP)	0.1	0.310	[11]

**Table 2:** Summary of methodological parameters reported in the included studies.

Author	Region	PAHs Detected (*n*)	Matrix Type	Extraction Method	Detection Method	LOD/LOQ (µg/kg)	Ref.
Al-Alam, J.	Lebanon	16 (9 detected)	Honey	QuEChERS + SPME	GC–MS/MS	LOD = 3 × S/N; LOQ = 10 × S/N (matrix-matched calibration)	[2]
Al-Alam, J.	Lebanon	16	Honey	QuEChERS + SPME	GC–MS/MS (ion-trap)	LOD: 0.00007–0.012; LOQ: 0.00023–0.040	[1]
Ciemniak, A.	Poland	23 (16 + additional)	Honey	Liquid–liquid extraction with n-hexane	GC–MS (HP 6890/5973, Agilent Technologies, Santa Clara, USA; SIM mode)	LOD: 0.022–0.109; LOQ: 0.066–0.329 (BaP: LOD 0.023, LOQ 0.07)	[3]
Derrar, S.	Algeria	13 (6 detected)	Honey	QuEChERS (MgSO_4_ + NaCl; d-SPE cleanup)	GC–MS/MS (Shimadzu GCMS-TQ8030, Shimadzu Corporation, Kyoto, Japan; EI mode, MRM)	LOD: 0.12–1.23; LOQ: 0.20–1.55 (compound-dependent)	[7]
Di Fiore, C.	Italy (Molise)	16	Honey, bees	Ultrasonication with dichloromethane/acetone (1:1 *v*/*v*), rotavapor, SPE cleanup	GC–IT/MS	NR	[22]
Ek-Huchim, J.P.	Mexico	16	Honey, *Apis mellifera*	Liquid–liquid extraction + clean-up (florisil, alumina, silica gel, Na_2_SO_4_)	GC–MS/MS (Thermo TSQ 8000 Evo, Thermo Fisher Scientific, Waltham, USA; SRM mode)	LOD: 0.009–0.099; LOQ: 0.02–0.26	[23]
Hungerford, N.L.	Australia (Queensland)	33	*Apis mellifera* honey (urban, peri-urban, rural, blends)	Solid-phase extraction (SPE)	GC–MS/MS (QHFSS method QIS34973)	LOQ: 0.5	[27]
Iwegbue, C.M.A.	Nigeria	16	Honey (*Apis mellifera*)	Ultrasonication with n-hexane and dichloromethane	GC-MS	NR	[9]
Jovetić, M.S.	Serbia	15	Honey, pollen	QuEChERS (with dSPE cleanup)	HPLC-FLD (Thermo Spectra System, Thermo Fisher Scientific, Waltham, USA; PAH C18 column)	LOD: <0.1–0.4 (e.g., BaA, Chr); LOQ: <0.2–2.0	[4]
Kamankesh, M.	Iran	16	Honey	High-density solvent-dispersive liquid–liquid microextraction (HDS/DLLME)	GC–MS (SIM mode)	LOD: 0.0003–0.0008; LOQ: 0.0009–0.0024	[19]
Kazazic, M.	Bosnia and Herzegovina	16 (9 detected)	Honey (*Apis mellifera*)	Ultrasonic bath extraction with n-hexane/acetone (1:1)	HPLC-UV/Vis (255 nm)	NR	[26]
Khatami, A.	Iran	4 (1 detected)	Honey (*Apis mellifera*)	Dispersive Solid Phase Extraction (DSPE) + Dispersive Liquid–Liquid Microextraction (DLLME) using Co–GA MOF and MDES	HPLC–DAD	LOD: 0.00018–0.00026; LOQ: 0.00060–0.00087	[18]
Lambert, O.	France	16	Honey, pollen, bee	Pressurized Liquid Extraction (ASE) + LLE (cyclohexane/ethyl acetate for honey) + SPE cleanup	GC–MS/MS (Triple Quadrupole System, Thermo Fisher Scientific, Waltham, USA)	LOD: 0.008–0.017; LOQ: 0.026–0.055	[28]
Mandelli, A.	Argentina	31 (16 PAHs + OPAHs + NPAHs)	Honey	SALLE (salting-out assisted liquid–liquid extraction)	UHPLC-(+)APCI-MS/MS	LOD: 0.00004–0.00977 µg/kg LOQ: NR	[8]
Marcolin, L.C.	Brazil	16	Stingless bee honey (*Meliponinae*)	QuEChERS	GC–MS/MS (Triple Quadrupole System, Agilent Technologies, Santa Clara, USA; SIM mode)	LOD 0.3–3; LOQ 1–10	[24]
Mohebbi, L.	Iran	16	Honey	Magnetic dispersive solid phase extraction (DSPE) + Solidification of floating organic droplet-based dispersive liquid–liquid microextraction (SFOD-DLLME)	GC–MS (Agilent 6890 GC + 5973 MS, HP-5 MS column; Agilent Technologies, Santa Clara, CA, USA)	LOD: 0.08–0.17; LOQ: 0.27–0.57	[29]
Murcia-Morales, M.	Austria, Denmark, Greece	27 (parent + alkylated PAHs)	Honey bees, pollen, propolis	QuEChERS	GC–MS/MS	iLOQ: 0.5–1; up to 5 for nitro-PAHs; values < iLOQ reported as trace	[20]
Ozoani, H.A.	Nigeria	16	Honey	Solvent extraction (acetone + dichloromethane, 50:50)	GC–FID (HP-5 column, dual detector; Agilent Technologies, Santa Clara, CA, USA; US EPA Method 8100)	LOD: <0.015; LOQ: 0.05	[21]
Passarella, S.	Italy	22 (EPA + additional)	Honey	Ultrasound–vortex assisted DLLME	GC–MS/MS (Thermo Trace 1310/TSQ 8000 Evo, SIM & full scan; Thermo Fisher Scientific, Waltham, MA, USA)	LOD: 0.003–0.029; LOQ: 0.009–0.095	[30]
Russo, M.V.	Italy	9	Honey	Ultrasound–Vortex Assisted DLLME (UVALLME),	GC–FID; GC–IT/MS (ion trap)	GC-FID: LOD 36–63; LOQ 41–74. GC-IT/MS: LOD 0.030–0.199; LOQ 0.069–0.466	[17]
Saitta, M.	Italy	16	Honey	QuEChERS	GC–MS/MS (Thermo Trace GC Ultra + TSQ Quantum XLS triple quadrupole, SRM mode; Thermo Fisher Scientific, Waltham, MA, USA)	LOD:0.10–5.21; LOQ: 0.33–17.19	[31]
Sari, M.F.	Turkey	16	Honey, pollen	Liquid–liquid extraction	GC–MS (Agilent 7890A/5975C; Agilent Technologies, Santa Clara, CA, USA)	NR	[32]
Sari, M.F.	Turkey	16	honey	Liquid–liquid extraction (MeOH, DCM), GPC + silica/alumina clean-up	GC–MS (SIM)	LOD: 0.0001–0.0044 µg/kg	[33]
Sawicki, T.	Poland	16	Honey, Bee bread, Bee pollen, Beeswax	QuEChERS	GC–MS (Ion Trap, SIM mode)	LOD: 0.08–0.26; LOQ: 0.24–0.78	[34]
Surma, M.	Europe-wide	16	Honey	QuEChERS	GC–MS (Varian 4000 Ion Trap, DB-5MS column, SIM mode; Varian Inc., Palo Alto, CA, USA)	LOD: 0.76–18.98 (ΣPAHs)	[25]
Toptanci, İ.	Turkey	16	Honey	QuEChERS	GC–MS/MS (Agilent 7890A + 7000B Triple Quadrupole, HP-5MS column, MRM mode; Agilent Technologies, Santa Clara, CA, USA)	LOD: 0.03–0.29; LOQ: 0.11–0.27	[35]
Wang, W.	China	16	Honey	Headspace-SPME	GC–MS (Agilent 7820A–5977E, SIM mode; Agilent Technologies, Santa Clara, CA, USA)	LOD: 0.00003–0.00019; LOQ: 0.00010–0.00063	[36]
Ayyildiz, E.G.	Turkey	16	Honey, bees	DCM/PE extraction + ultrasound	GC–MS (Agilent 7890A/5975C, SIM mode, HP-5MS column; Agilent Technologies, Santa Clara, CA, USA)	NR	[37]
Antwi-Boasiako, S.	Ghana	14	Honey	QuEChERS (ACN + MgSO_4_/NaOAc; d-SPE cleanup with PSA/C18/MgSO_4_)	HPLC–FLD	LOD: 0.10–6.46; LOQ: NR	[38]

Abbreviations: BaP, benzo[a]pyrene; BaA, benzo[a]anthracene; BbF, benzo[b]fluoranthene; BkF, benzo[k]fluoranthene; Chr, chrysene; DahA, dibenzo[a,h]anthracene; IcdP, indeno[1,2,3–cd]pyrene; Phe, phenanthrene; Pyr, pyrene; FL, fluoranthene; Naph, naphthalene; etc.

**Table 3:** Summary of Detected PAHs and Their Concentrations in Honey Across 29 Studies.

Author	Region	PAHs Detected (*n*)	Honey Type	Individual PAH Concentration (µg/kg)	ΣPAHs (µg/kg, Mean/Range)	Max PAH (Compound, µg/kg)	Ref.
Al-Alam, J.	Lebanon	16 (9 detected)	NR	Naph: 33.3; Ace: 25.3; FLu: 33.03; Phe: 10.33; Ant: 15.87; FL: 20.2; Pyr: 14.7; BaA: 8.74; Chr: 5.36	Mean 166.83; Range 5.36–33.03	Naph, 33.03	[2]
Al-Alam, J.	Lebanon	16	Multifloral	BaP: 0.17; Naph: <LOQ–1.71; most PAHs <LOQ–0.56	Range < LOQ–6.05	Naph, 1.71	[1]
Ciemniak, A.	Poland	23 (16 + additional)	Multifloral	BaP: <0.005–0.024	Range < 0.005–0.311 (up to 0.311)	NR, 0.076	[3]
Derrar, S.	Algeria	13 (6 detected)	Mixed: Unifloral & Multifloral	FLu: 5.73; Phe: 2.33; Ant: 1.55; BaA; Chr; Acy	NR	FLu, 5.73	[7]
Di Fiore, C.	Italy (Molise)	16	Multifloral	BaP: 0.19–0.38; FL: 0.34–2.20; Chr: 0.27–0.62	Range 2.48–9.58	FL, 2.20	[22]
Ek-Huchim, J.P.	Mexico	16	NR	FLu: 2.1–6.2; Phe: 1.3–4.2; Ant: 1.4–3.1; Pyr: 1.9–4.6; BaP: 0.3–0.9	Range 3.2–14.6	FLu, 6.2	[23]
Hungerford, N.L.	Australia (Queensland)	33	Multifloral	BaP: 0.0079; Chr: 0.0049; FL: 0.0109	Range 0.0102–0.0297	FL, 0.0109	[27]
Iwegbue, C.M.A.	Nigeria	16	Multifloral	NW: BaP: 1.83–3.26; DahA: 2.66–5.29; SW: BaP: 1.92–2.73; DahA: 3.27–5.85; SE: BaP: 2.15–4.32; DahA: 4.17–6.86; ND: BaP: 4.65–7.91; DahA: 6.24–11.4	Range NW: 17.46–22.97; SW: 22.07–29.64; SE: 26.32–35.51; ND: 37.62–47.15	DahA, 11.4	[9]
Jovetić, M.S.	Serbia	15	Multifloral	BaP: 0.53;	Range 2.8–18	Naph, ≈5	[4]
Kamankesh, M.	Iran	16	Mixed: Unifloral & Multifloral	Min: 1.22; Max: 11.63; Mean: ≈4.6	Mean ≈ 4.6; Range 1.22–11.63	NR, 11.63	[19]
Kazazic, M.	Bosnia and Herzegovina	16 (9 detected)	Multifloral	BaP: ND–6.12 (most samples ND; one sample 6.12)	Range 2.68–12.58 (in 4 positive samples)	BaP, 6.12	[26]
Khatami, A.	Iran	4 (1 detected)	Mixed: Unifloral & Multifloral	Pyr: 0.00556–0.01298	Range 0.00556–0.01298	Pyr, 0.01298	[18]
Lambert, O.	France	16	Multifloral	ND–0.155	Range ND–0.155	NR, 0.155	[28]
Mandelli, A.	Argentina	31 (16 PAHs + OPAHs + NPAHs)	Multifloral	Parent PAHs: ND–91.5; OPAHs: ND–103; NPAHs: ND–22.1	Range ND–103 (across all PAH classes)	OPAHs, 103	[8]
Marcolin, L.C.	Brazil	16	NR	1.4–23.3	Range 1.4–23.3	NR, 23.3	[24]
Mohebbi, L.	Iran	16	NR	ND–118.25	Range ND–118.25	IcdP, 118.25	[29]
Murcia-Morales, M.	Austria, Denmark, Greece	27 (parent + alkylated PAHs)	NR	ND–7.67	Range ND–7.67	Chr, 7.67	[20]
Ozoani, H.A.	Nigeria	16	Multifloral	0.439–3.22	2.51–3.08	PAH4, 3.22	[21]
Passarella, S.	Italy	22 (EPA + additional)	Multifloral	0.003–5.91	NR	FL, 5.91	[30]
Russo, M.V.	Italy	9	Mixed: Unifloral & Multifloral	<LOQ–38.7	Range < LOQ–38.7	NR, 38.7	[17]
Saitta, M.	Italy	16	Mixed: Unifloral & Multifloral	0.11–16.0	Mean 0.16; Range 0.11–16.0	NR, 16	[31]
Sari, M.F.	Turkey	16	Multifloral	0.10–5.19	Mean 1.71; Range 0.10–5.19	BaP, 0.45	[32]
Sari, M.F.	Turkey	16	Multifloral	NR	Range 1.22–11.63	NR, 11.63	[33]
Sawicki, T.	Poland	16	Multifloral	0.02–16.0	Mean 1.03; Range 0.02–16.0	BaP (in propolis), 3.58	[34]
Surma, M.	Europe-wide	16	Multifloral	Phe up to 1.19	Mean 1.96; Range 0.14–5.03	Phe, 1.19	[25]
Toptanci, İ.	Turkey	16	Multifloral	Urban: 3.81–16.14; Rural: 2.27–7.24	Urban: Mean 10.12; Range 3.81–16.14; Rural: Mean 4.11; Range 2.27–7.24	Σ16PAHs (urban), 16.14	[35]
Wang, W.	China	16	Unifloral	1.22–11.63	Range 1.22–11.63	NR, 11.63	[36]
Ayyildiz, E.G.	Turkey	16	NR	Individual PAHs: 0.0249–0.3809	Ovaakca: 380.87; Cumalikizik: 217.96 (µg/kg dw)	ΣPAHs (Bee Ovaakca, dw), 380.87	[37]
Antwi-Boasiako, S.	Ghana	14	Multifloral	Mean: 1.29. Range: 0.10–5.80	Mean 1.29; Range 0.10–5.80	NR, 5.80	[38]

Abbreviations: EDI, Estimated Daily Intake; HQ, Hazard Quotient; HI, Hazard Index; ILCR, Incremental Lifetime Cancer Risk; TEQ, Toxic Equivalency; DDI, Daily Dietary Intake; ECR, Excess Cancer Risk; BaP, Benzo[a]pyrene.

**Table 4:** Risk assessment of PAHs in honey across different studies.

Study (Author, Year)	Country/Region	Matrix	Risk Assessment Method(s)	Risk Characterization	Ref.
Derrar, S. (2024)	Algeria	Honey	EDI, HQ	Acceptable Risk (HQ < 1, EDI low)	[7]
Iwegbue, C.M.A. (2016)	Nigeria	Honey	ILCR, TEQ, BaP threshold	Moderate Risk (BaP > 1 µg/kg)	[9]
Jovetić, M.S. (2018)	Serbia	Honey & bee Products	BaP threshold (EU/Serbia)	Below threshold (BaP < 1 µg/kg)	[4]
Kamankesh, M. (2022)	Iran	Honey	EDI, HI, ILCR	Some exceed EU BaP limits; overall moderate risk	[19]
Marcolin, L.C. (2023)	Brazil	Honey & bee Products	TEQ_BaP_, DDI, ECR	23% exceeded dietary limits (BaP–TEQ > threshold)	[24]
Ozoani, H.A. (2020)	Nigeria	Honey	BaP threshold (EU)	Below threshold	[21]
Sari, M.F. (2022)	Turkey	Honey	ILCR (adults, US–EPA model))	Low Risk	[33]
Sawicki, T. (2023)	Poland	Honey	HQ (children)	No significant risk	[34]
Ayyildiz, E.G (2019)	Turkey	Honey & bee tissue	TEQ (Bee tissue)	Data not directly for honey	[37]
Antwi-Boasiako, S (2017)	Ghana	Honey	ILCR (USEPA model)	Acceptable Risk (ILCR < 10^−6^–10^−5^)	[38]

Abbreviations: EDI, Estimated Daily Intake; HQ, Hazard Quotient; HI, Hazard Index; ILCR, Incremental Lifetime Cancer Risk; TEQ, Toxic Equivalency; DDI, Daily Dietary Intake; ECR, Excess Cancer Risk; BaP, Benzo[a]pyrene.

**Table 5:** Carcinogenic risk estimates of PAHs in honey across 29 studies, based on TEQ_BaP_ and MEQ_BaP_.

Study	Country	BaA	BaP	BbF	BkF	Chr	DahA	IndP	TEQ_BaP_	MEQ_BaP_	BaP_EQ_ –TEQ	BaP_EQ_ –MEQ	CR– TEQ	CR–MEQ	Risk Level	Ref.
Al–Alam, J.	Lebanon	0.44	0.37	0.88	0.61	0.34	0.21	0.57	0.78	0.94	1.91 × 10^1^	2.31 × 10^1^	1.40 × 10^−1^	1.69 × 10^−1^	Risk	[2]
Al–Alam, J.	Lebanon	0	0.17	0	0	0	0	0	0.17	0.17	4.19 × 10^0^	4.19 × 10^0^	3.06 × 10^−2^	3.06 × 10^−2^	Risk	[1]
Ciemniak, A.	Poland	0.07	0.06	0.05	0.04	0.06	0.4	0.05	0.48	0.22	1.18 × 10^1^	5.31 × 10^0^	8.59 × 10^−2^	3.87 × 10^−2^	Risk	[3]
Derrar, S.	Algeria	1.41	2.16	0.68	0.17	0.3	0	0	2.37	2.47	6.10 × 10^−5^	6.35 × 10^−5^	4.45 × 10^−4^	4.64 × 10^−4^	Acceptable	[7]
Di Fiore, C.	Italy (Molise)	0.19	0.14	0.04	0.02	0.05	0.01	0.01	0.17	0.17	4.30 × 10^0^	4.31 × 10^0^	3.14 × 10^−2^	3.14 × 10^−2^	Risk	[22]
Ek-Huchim, J.P.	Mexico	0.05	0.1	0	0.01	0.04	0	0.02	0.11	0.11	2.64 × 10^0^	2.76 × 10^0^	1.93 × 10^−2^	2.02 × 10^−2^	Risk	[23]
Hungerford, N.L.	Australia (Queensland)	0.006	0.017	0.003	0.002	0.004	0	0.006	0.02	0.02	4.57 × 10^−1^	5.03 × 10^−1^	3.33 × 10^−3^	3.67 × 10^−3^	Risk	[27]
Iwegbue, C.M.A.	Nigeria	1.22	7.91	3.94	1.76	2.19	0.96	4.41	9.85	10.87	2.43 × 10^2^	2.68 × 10^2^	1.77 × 10^0^	1.96 × 10^0^	Risk	[9]
Jovetić, M.S.	Serbia	0.07	0.1	0.22	0.11	0.06	0.17	0.06	0.31	0.24	7.55 × 10^0^	5.96 × 10^0^	5.51 × 10^−2^	4.35 × 10^−2^	Risk	[4]
Kamankesh, M.	Iran	0	0	0	0	0	0	0	0.00	0.00	0.00 × 10^0^	0.00 × 10^0^	0.00 × 10^0^	0.00 × 10^0^	Safe	[19]
Kazazic, M.	Bosnia and Herzegovina	0.44	6.12	0.05	0.02	0.2	0.05	2.56	6.48	6.98	1.60 × 10^2^	1.72 × 10^2^	1.17 × 10^0^	1.26 × 10^0^	Risk	[26]
Khatami, A.	Iran	0	0	0	0	0	0	0	0.00	0.00	0.00 × 10^0^	0.00 × 10^0^	0.00 × 10^0^	0.00 × 10^0^	Safe	[18]
Lambert, O.	France	0	0	0	0	0	0	0	0.00	0.00	0.00 × 10^0^	0.00 × 10^0^	0.00 × 10^0^	0.00 × 10^0^	Safe	[28]
Mandelli, A.	Argentina	0	0	0	0	0	0	0	0.00	0.00	0.00 × 10^0^	0.00 × 10^0^	0.00 × 10^0^	0.00 × 10^0^	Safe	[8]
Marcolin, L.C.	Brazil	0.555	0.205	0.74	0.46	0.46	0.14	0.475	0.53	0.68	1.30 × 10^1^	1.68 × 10^1^	9.49 × 10^−2^	1.23 × 10^−1^	Risk	[24]
Mohebbi, L.	Iran	0	0	0	0	0	0	0	0.00	0.00	0.00 × 10^0^	0.00 × 10^0^	0.00 × 10^0^	0.00 × 10^0^	Safe	[29]
Murcia-Morales, M.	Austria, Denmark, Greece	0.705	0.585	0.645	0.435	0.795	0	0	0.73	0.87	1.79 × 10^1^	2.13 × 10^1^	1.31 × 10^−1^	1.56 × 10^−1^	Risk	[20]
Ozoani, H.A.	Nigeria	0	0	0	0	0	0	0	0.00	0.00	0.00 × 10^0^	0.00 × 10^0^	0.00 × 10^0^	0.00 × 10^0^	Safe	[21]
Passarella, S.	Italy	0	0	0	0	0	0	0	0.00	0.00	0.00 × 10^0^	0.00 × 10^0^	0.00 × 10^0^	0.00 × 10^0^	Safe	[30]
Russo, M.V.	Italy	0	0	0	0	0	0	0	0.00	0.00	0.00 × 10^0^	0.00 × 10^0^	0.00 × 10^0^	0.00 × 10^0^	Safe	[17]
Saitta, M.	Italy	0	0	0	0	0	0	0	0.00	0.00	0.00 × 10^0^	0.00 × 10^0^	0.00 × 10^0^	0.00 × 10^0^	Safe	[31]
Sari, M.F.	Turkey	0.2665	0.099	0.1045	0.0845	0.1795	0.0635	0.0785	0.21	0.20	5.14 × 10^0^	4.98 × 10^0^	3.75 × 10^−2^	3.64 × 10^−2^	Risk	[32]
Sari, M.F.	Turkey	0.174	0.067	0.0825	0.061	0.061	0.045	0.123	0.15	0.16	3.71 × 10^0^	3.97 × 10^0^	2.71 × 10^−2^	2.89 × 10^−2^	Risk	[33]
Sawicki, T.	Poland	0.115	0.1	0.145	0.08	0.095	0	0	0.13	0.16	3.13 × 10^0^	3.85 × 10^0^	2.28 × 10^−2^	2.81 × 10^−2^	Risk	[34]
Surma, M.	Europe–wide	0.315	0.145	0.275	0.195	0.23	0	0	0.21	0.26	5.08 × 10^0^	6.53 × 10^0^	3.71 × 10^−2^	4.77 × 10^−2^	Risk	[25]
Toptanci, İ.	Turkey	0.94	0.52	1.34	0.59	0.865	0	0	0.75	1.01	1.86 × 10^1^	2.49 × 10^1^	1.36 × 10^−1^	1.82 × 10^−1^	Risk	[35]
Wang, W.	China	0.575	0.515	0.79	0	0.625	0	0	0.65	0.77	1.61 × 10^1^	1.90 × 10^1^	1.17 × 10^−1^	1.39 × 10^−1^	Risk	[36]
Ayyildiz, E.G.	Turkey	0	0	0	0	0	0	0	0.00	0.00	0.00 × 10^0^	0.00 × 10^0^	0.00 × 10^0^	0.00 × 10^0^	Safe	[37]
Antwi-Boasiako, S.	Ghana	0	0.18	0	0	0	0	0	0.18	0.18	4.44 × 10^0^	4.44 × 10^0^	3.24 × 10^−2^	3.24 × 10^−2^	Risk	[38]

All concentrations are expressed in µg/kg. Abbreviations: BaA, Benzo[a]anthracene; BaP, Benzo[a]pyrene; BbF, Benzo[b]fluoranthene; BkF, Benzo[k]fluoranthene; Chr, Chrysene; DahA, Dibenzo[a,h]anthracene; IndP, Indeno[1,2,3–cd]pyrene; TEQ, Toxic Equivalent; MEQ, Mutagenic Equivalent; BaP_EQ_, BaP Equivalent; CR, Cancer Risk. Note: TEQ_BaP_ and MEQ_BaP_ values were calculated using seven EPA priority PAHs with established toxic equivalency factors (TEFs) or mutagenicity equivalency factors (MEFs): BaA, BaP, BbF, BkF, Chr, DahA, and IndP.

## Data Availability

No new data were created or analyzed in this study.

## Abbreviations

The following abbreviations are used in this manuscript:

Ace	Acenaphthene
Acy	Acenaphthylene
Ant	Anthracene
BaA	Benzo[a]anthracene
BaP	Benzo[a]pyrene
BaP_EQ_	Benzo[a]pyrene Equivalent
BbF	Benzo[b]fluoranthene
BkF	Benzo[k]fluoranthene
Chr	Chrysene
CR	Cancer Risk
DahA	Dibenzo[a,h]anthracene
DDI	Daily Dietary Intake
EDI	Estimated Daily Intake
EFSA	European Food Safety Authority
EPA 16	The USEPA list of 16 priority PAHs
EU	European Union
ECR	Excess Cancer Risk
FL	Fluoranthene
FLu	Fluorene
GC–FID	Gas Chromatography–Flame Ionization Detection
GC–MS	Gas Chromatography–Mass Spectrometry
GC–MS/MS	Gas Chromatography–Tandem Mass Spectrometry
HI	Hazard Index
HQ	Hazard Index
HPLC	High Performance Liquid Chromatography
IcdP	Indeno[1,2,3–cd]pyrene
ILCR	Incremental Lifetime Cancer Risk
LC–MS/MS	Liquid Chromatography–Tandem Mass Spectrometry
LOD	Limit of Detection
LOQ	Limit of Quantification
MEF	Mutagenic Equivalency Factor
MEQ_BaP_	Mutagenic Equivalent concentration relative to BaP
ND	Not Detected
NPAHs	Nitrated Polycyclic Aromatic Hydrocarbons
NR	Not Reported
OPAHs	Oxygenated Polycyclic Aromatic Hydrocarbons
PAH4	Sum of BaP, BbF, BaA, and Chr (EU indicator)
PAHs	Polycyclic Aromatic Hydrocarbons
Phe	Phenanthrene
PRISMA	Preferred Reporting Items for Systematic Reviews and Meta–Analyses
Pyr	Pyrene
QuEChERS	Easy, Cheap, Effective, Rugged, and Safe (sample preparation)
ΣPAHs	Sum of Polycyclic Aromatic Hydrocarbons
SPME	Solid-Phase Microextraction
TEF	Toxic Equivalency Factor
TEQ_BaP_	Toxic Equivalent concentration relative to BaP
USEPA	United States Environmental Protection Agency
WHO	World Health Organization
dw	Dry Weight
